# Unleashing the potential of catalytic RNAs to combat mis-spliced transcripts

**DOI:** 10.3389/fbioe.2023.1244377

**Published:** 2023-11-16

**Authors:** Bashayer A. Khalifah, Shareefa A. Alghamdi, Ali H. Alhasan

**Affiliations:** ^1^ Institute for Bioengineering, Health Sector, King Abdulaziz City for Science and Technology (KACST), Riyadh, Saudi Arabia; ^2^ Faculty of Sciences, King Abdulaziz University, Jeddah, Saudi Arabia; ^3^ College of Science and General Studies, Alfaisal University, Riyadh, Saudi Arabia

**Keywords:** intron retention, synthetic biology, spliceosome, spliceopathies, catalytic RNAs

## Abstract

Human transcriptome can undergo RNA mis-splicing due to spliceopathies contributing to the increasing number of genetic diseases including muscular dystrophy (MD), Alzheimer disease (AD), Huntington disease (HD), myelodysplastic syndromes (MDS). Intron retention (IR) is a major inducer of spliceopathies where two or more introns remain in the final mature mRNA and account for many intronic expansion diseases. Potential removal of such introns for therapeutic purposes can be feasible when utilizing bioinformatics, catalytic RNAs, and nano-drug delivery systems. Overcoming delivery challenges of catalytic RNAs was discussed in this review as a future perspective highlighting the significance of utilizing synthetic biology in addition to high throughput deep sequencing and computational approaches for the treatment of mis-spliced transcripts.

## Graphical Abstract

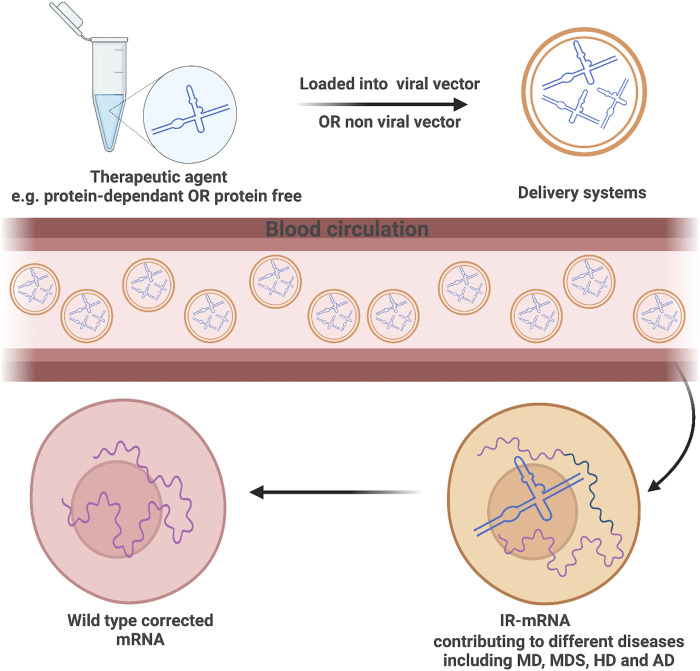

## Introduction

Various pathogenesis could result from spliceopathies, in which pre-mRNA undergoes a dysregulated-splicing process. Spliceosomes are the largest ribonucleoproteins that assemble around newly synthesized RNA transcripts in order to perform two distinctive trans-esterification reactions, which contribute to the precise removal of the intervening non-coding sequences (introns) followed by joining the coding regions (exons) during the transcription maturation step (Valadkhan and Manley, 2001). Along with the two-post transcriptional modification; 5′ capping and 3′ poly A tailing, splicing occurs in a highly coordinated manner to produce a fully functional messenger RNA (mRNA) ready to be translated into the protein of interest via the cytoplasmic ribosomes. Mammalian spliceosomes consist of five essential uridine-rich small nuclear RNAs (U1, U2, U4, U5 and U6 snRNAs), which recruit a massive number of auxiliary splicing factors (∼200 proteins) needed for initiating the splicing reaction (Rappsilber et al., 2002; Valadkhan et al., 2009; Suñé-Pou et al., 2020). Spliceosomes drive the alternative splicing (AS), which is a critical regulatory process contributing to the vast diversity of translated proteins in mammalian cells. For a given transcript, there are five major AS events that might take place; constitutive splicing, exon skipping pattern, mutually exclusive exons, alternative 5′ or 3′ splice site events (Graveley, 2001; Modrek and Lee, 2002; Yeo et al., 2005) and intron retention (IR) (Galante et al., 2004; Edwards et al., 2016; Rekosh and Hammarskjold, 2018).

Within the IR event, transcripts harbor two or more introns that failed to be spliced out from the mature mRNA due to spliceosomal dysfunction leading to spliceopathies (Monteuuis et al., 2019). Among all AS events, IR received a minimal attention and considered to be a rare event in which hydrolysis often occurs via the cellular degradation pathway. However, growing number of reports suggest that IR could affect 80% of the coding genes (Middleton et al., 2017), especially those involved in cell differentiation (Llorian et al., 2016) and cell cycling (Braunschweig et al., 2014; Llorian et al., 2016; Middleton et al., 2017). Per the cancer genomic atlas (TSGA) and the transcriptomic cancer studies, IR is considered the common AS mode among all cancer types as it accounts for the wide diversities in cancer transcriptomes (Supek et al., 2014; Dvinge and Bradley, 2015).

Interestingly, spliceosomal dysfunction is often caused by errors in the transesterification reactions of the cis-acting elements and/or trans-acting factors during the spliceosomal assembly. Chemically, transesterification is a type of SN2 nucleophilic substitution reactions where synchronously one of the ester bonds is broken and another ester bond is formed. In a typical splicing reaction, two consecutive reaction takes place in the nucleus (nuclear splicing) as follows: first, nucleophilic attack of the hydroxyl group at 2′ carbon atom of the branched adenosine located in the introns will results in releasing the first free 5′exon and 2′-5′ unusual phosphodiester bond formation between the hydroxyl group of the branched adenosine and 5′ phosphoryl group of the 5′ end of intron to form partial lariat structure in step commonly known as branching. Second step known as ligation which involves the cleavage at 3′ splice site done by the attack of the 3′ hydroxyl group of the 5′ exon and leads to joining of the exons together and release the intron (Horowitz and Abelson, 1993; Shi et al., 2018).

Consequently, mutations in both cis-acting elements and trans-acting factors could inevitably influence the functionality of spliceosome machinery leading to spliceosomopathies. For instance, alteration in the cis-acting elements such as enhancers and silencers significantly affects the catalytic reaction leading to mis-splicing (Scotti and Swanson, 2016; Anna and Monika, 2018). IR that results from mutations in the trans-acting factors can disrupt the activity of spliceosomes as well. For example, mutations occur in the most important component factors expressed in spliceosome (PRPF31, PRPF3 and PRPF8) lead to hereditary disease in the eye called retinitis pigmentosa (RP) that dramatically delayed the spliceosome assembly affecting the pre-mRNA splicing. Such mutations decreased the removal of ∼9% of the introns from coding genes not only from the retina of the eye, but other tissues such as lymphoblast (Tanackovic et al., 2011). Other mutations affecting multiple E/A splicing complex, namely, U2AF35, ZRSR2, SRSF2 and SF3B1, lead to myelodysplastic syndromes, which is a heterogeneous group of myeloid neoplasms that manifests bone marrow failure leading to acute myeloid leukemia (Yoshida et al., 2011). Another example of mutations in three important spliceosomal maintenance proteins (TDP-43, FUS/TLS, and SMN) cause profound loss of the spliceosomal integrity and lead to amyotrophic lateral sclerosis (ALS) and spinal muscular atrophy (SMA) (Tsuiji et al., 2013). Even though it is widely accepted that exons skipping during the splicing process is considered the most common patterns of AS and account for ∼60% of spliced transcripts (Sugnet et al., 2003; Dvinge and Bradley, 2015) it was reported in multiple cancer genomic studies that IRs were predominant in all analyzed cancer transcripts (Dvinge and Bradley, 2015). Four decades ago, when IR is discovered, it revealed new insights of its role in regulation of gene expression, pathogenesis, and treatment approaches (Kumari et al., 2022). Researchers have been captivated in carrying out pre-clinical and clinical trials on new drug molecules to either interfere, inhibit, or alter the spliceosome itself or the splicing reactions to treat various spliceopathies (Rupaimoole and Slack, 2017; Bonnal et al., 2020; Desterro et al., 2020; Steensma et al., 2021; Childs-Disney et al., 2022; Murphy et al., 2022; Qin et al., 2022; Suresh et al., 2022; Zhu et al., 2022; Velema and Lu, 2023). It is worth noting that IR can be used as a diagnostic biomarker for the intronic expansion disorders in addition to its applications for therapeutic purposes (Sznajder et al., 2018). Yet, the field of IR is still emerging and there is more to explore (Vanichkina et al., 2018).

## The fate of intron containing transcripts

**FIGURE 1:**
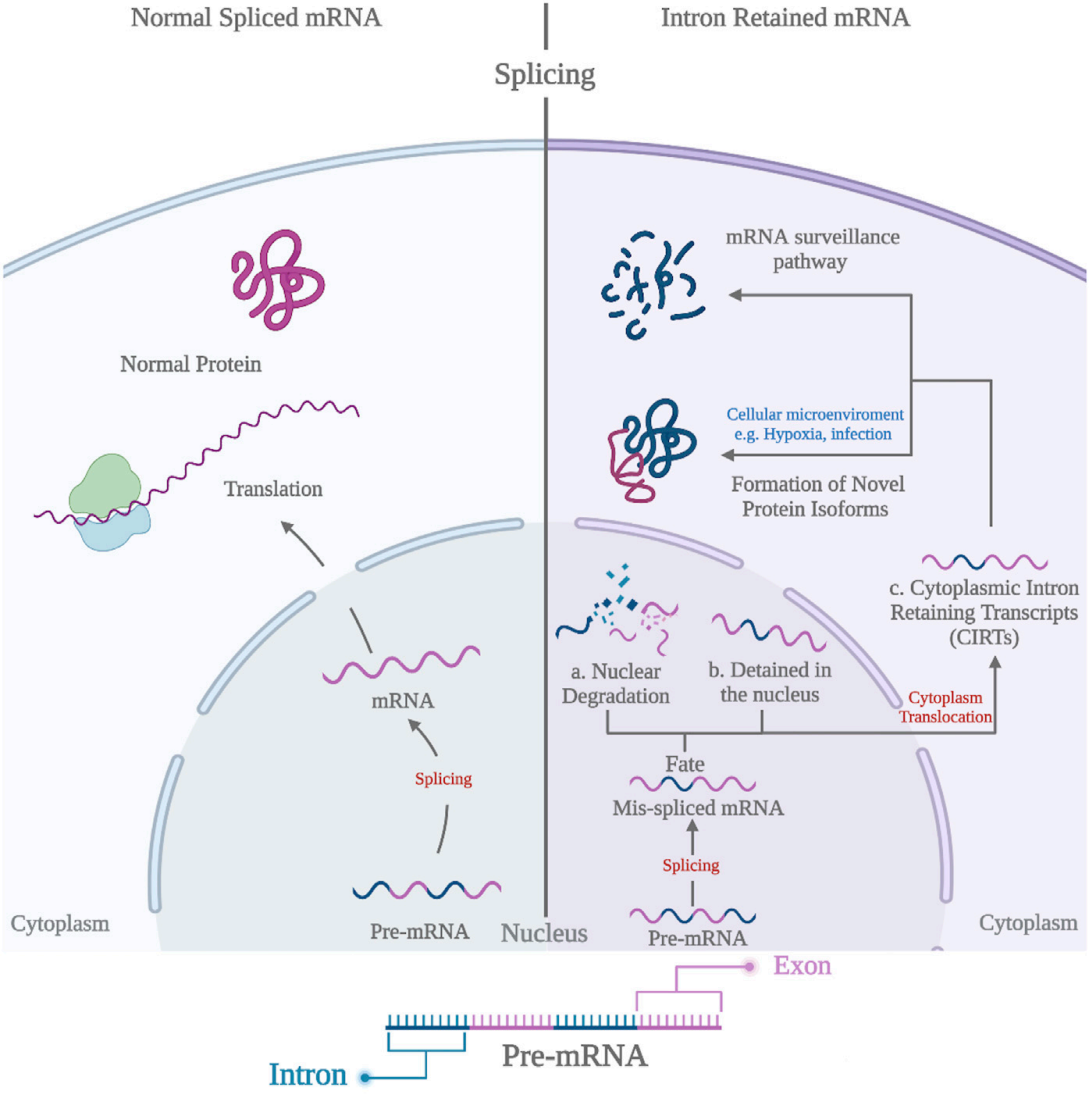
Different Fates of Pre-mRNA transcripts. A). Nuclear degradation B). Detained Introns (DIs) C). Cytoplasmic intron retaining transcripts (CIRTs).

Mammalian systems exert diverse regulatory processes to control the fate of IR-containing mRNA transcripts (IR-mRNAs) (Figure 1), which are often subjected to nuclear retention accompanied with nuclear degradation via the exosomal degradation pathway (Gudipati et al., 2012). However, a novel class of introns termed Detained Introns (DIs) was discovered recently in which introns are retained within the nucleus and protected from degradation, yet exhibiting a slower splicing process than other introns within the same gene (Figure 1) (Boutz et al., 2015; Mauger et al., 2016; Naro et al., 2017). Interestingly, incomplete transcripts might be coupled with exporting proteins and translocated to the cytoplasm to form the Cytoplasmic Intron Retaining Transcripts (CIRTs) (Figure 1) (Yap et al., 2012; Buckley et al., 2014). CIRTs can be degraded in the cytoplasm via the mRNA surveillance pathways and considered to be important check points to remove mis-spliced mRNAs (Powers et al., 2020). Surveillance pathways include 1) the non-sense mediated decay (NMD) pathway, which is triggered by the presence of the premature termination codon (PTC) in CIRTs (Lejeune and Maquat, 2005; Jaillon et al., 2008; Wong et al., 2013; Rekosh and Hammarskjold, 2018), 2) the no-go decay pathway activated in the presence of stalled ribosomes (Passos et al., 2009), and 3) the non-stop decay pathway targeting the degradation of transcripts that lack PTC (Van Hoof et al., 2002; Vasudevan et al., 2002). Evidently, CIRTs can escape these mRNA surveillance pathways and proceed to produce novel protein isoforms (Gontijo et al., 2011; Yap et al., 2012; Nasif et al., 2018). Experimental validation showed that miR-128 has the ability to suppress the NMD factors (UPF1 and MLN51) leading to IR-mRNAs escape followed by the production of protein isoforms (Bruno et al., 2011). Strikingly, the ability of CIRTs to avoid the NMD pathway depends on the cellular micro-environmental conditions such as hypoxia, infection, and the lack of nutrients (Karam et al., 2013; Hug et al., 2015; Li et al., 2017; Nasif et al., 2018). Those conditions of cellular microenvironment are well-established hallmarks for a wide range of inflammatory-based diseases ranging from cancer to neuropathies, which emphasize the significance of IR in pathological states (Brady et al., 2017; Farina et al., 2020; Massonneau et al., 2020; Tan et al., 2020).

The survival of IR-mRNAs from the cellular regulatory control becomes more apparent due to the advancement in IR-mRNA detection methods such as deep sequencing (Zheng et al., 2020). In a similar manner, the biological role of IR-mRNAs in both physiological and pathological states appears of high importance owing to the advancement of computational analyses (Jacob and Smith, 2017; Grabski et al., 2021). The power of computational technology and bioinformatics has been employed to develop programs capable to spot the intron retaining transcripts with high speed, accuracy, and sensitivity while building a huge IR library database that could be used as a reference for future IR studies as summarized in Table 1 (Bai et al., 2015; Middleton et al., 2017; Li et al., 2020). Apparently, current algorithms that detect AS in general might be tweaked to specifically detect IR (Zheng et al., 2020). Recent evolution of the deep tech and artificial intelligence have enormously improved the outcome for the precise and accurate detection of intronic sequences among transcripts to assist in the diagnosis of intronic abnormalities and aberrant splicing events (Zheng et al., 2020). For instance, Sun et al. (2023) employ the Intron Retention Index (IRI), an IRtools that provides IR analysis reads from RNA sequences collected from patients with systemic lupus erythematosus (SLE). The study reported dysregulation in IR as a hallmark of SLE disorder, which can be incorporate to enhance the accuracy of the IR detection. DeepRetention has the ability to predict the depth in intronic regions through pattern modelling, take the sequence depth into account as its main input to provide more detailed and accurate detection data like the intron length and the likelihood of retained introns (Wu et al., 2023).

**TABLE 1:** Current IR detection tool.

Tool name	Abbreviation	Detection method	Advantages	Limitation	References
Intron Retention call	IRcall	Uses ranking strategy to calculate IR score	- Reduce false positive results	- It depends on the quality of the used alignment tool to collect data	Bai et al. (2015)
Intron Retention classifier	IRclassifier	Uses machine learning technology to build up random forest to detect IR events	- High precision		
			- Identification of both known and novel IR events		
Intron Retention Finder	IRFinder	It detects IR events using IR ratio via measuring the intronic abundance and splicing level	fast and sensitive detection	Possible overlapping between introns and exons from other transcripts	Middleton et al. (2017)
			High Accuracy and precision	Calculating IR based on junction reads not on the expression level of intron	
			Free available database for over 2000 IR human samples	Multiple position reads from the genome produces noise in the results	
			Efficient detecting of low coverage. IR events		
Intron REtention Analysis and Detector	iREAD	Employs the entropy score to determine the distribution of intronic reads across the intron region	Limited exon-intron overlapping during read	It has no differential analysis	Li et al. (2020)
			Analyze both splice junction reads and intron expression level		
			Flexible running operating system		
			Sensitive		
Keep Me Around	KMA	R packaging tool to quantify IR in RNA data	Reduced false positive results by combining replicates	The IR analysis and quantification are performed in different software	Pimentel et al. (2015)
				The common feature of retained intron which is flat distribution is not identified	

In fact, the current algorithms lack the ability to annotate IR-containing mRNAs hindering the build up of a database parallel to that of intron-containing genomic DNAs, along with eliminating the differential expressions of IR-containing mRNAs. To overcome such limitations, advanced *in vivo* cell-imaging techniques have been successfully implemented to detect the presence and the expression levels of IR-containing mRNAs where non-invasive bioluminescence reporters are used to screen the IR splicing events while offering real time quantification (Shi et al., 2018; Zheng et al., 2019; Xie et al., 2020). Combining *in silico* computational methods with *in vivo* imaging techniques could provide accurate and reliable outcomes to ensure greater impacts in terms of detection and visualization. While detection techniques can bring the scientific community one step closer to rescuing patients from the deleterious effects of IR mis-spliced transcripts, they need to be coupled with therapeutic interventions to strengthen the battle against the intron-causing diseases. The field of manipulating and treating IR is still in its infancy since the number of related studies is limited, which warrants the need for extensive investigations.

## Protein-free snRNAs as catalytic RNAs

The increased demands to treat spliceopathies have ignited the development of innovative therapeutic approaches such as spliceosome-mediated RNA *trans*-splicing (SMaRT) (Wally et al., 2012), splice switching oligonucleotides (SSO) (Havens and Hastings, 2016), CRISPR/Cas9 (Yuan et al., 2018) and nanomedicine (Garcia-Blanco, 2003; Havens et al., 2013; Suñé-Pou et al., 2017; Suñé-Pou et al., 2020). For decades, scientists have been in route to develop treatments for mis-spliced transcripts using different re-engineered genetic tools such as group I introns ribozymes. *In vitro* studies showed that these ribozymes can be re-engineered to employ the trans-splicing type of reaction in order to repair the mis-spliced transcripts and generate a functioning protein having high specificity and fidelity (Sullenger and Cech, 1994; Watanabe and Sullenger, 2000; Ryu et al., 2003; Song and Lee, 2006). Nonetheless, ribozymes can recognize one splicing site and replace the defective part at either 5′ or 3’ ends. Amini et al investigated the development of a ribozyme that resembles human spliceosomes in recognizing two splicing sites, excising introns, and joining the two flanking exons. This novel spliceozyme showed a significant removal of 100 nucleotides from the intron of interest followed by the production of a functioning protein with high accuracy (Amini et al., 2014; Amini and Müller, 2015), which is potentially feasible for a wide range of therapeutic applications.

Valadkhan et al. developed a small spliceozyme to perform splicing reactions by using the mammalian catalytic core of the spliceosomes, U2 and U6 snRNA, to efficiently catalyze an *in vitro* intron removal via resembling the first two trans-esterification splicing reactions in the nucleus. The results showed the successful removal of introns and subsequent ligation of exons from synthetic oligonucleotides constructs forming IR-free RNA products (Valadkhan and Manley, 2001; Valadkhan et al., 2009). This could unleash the potential of protein-free catalytic RNAs as artificial spliceozymes in hopes to expedite their translation into clinics via acquiring engineered delivery systems to enhance their efficacy *in vivo*. Many pitfalls and challenges will need to be overcome prior to the *in vivo* testing of spliceozymes including preserving the stability of the protein-free catalytic RNAs against the degradative enzymes present in serum, minimizing immunogenicity, overcoming biological membranes, and maximizing the efficacy of the splicing reaction upon introduction to the target cells carrying mis-spliced transcripts.

## Overcoming delivery challenges of catalytic RNAs

Synthetic biology has served the scientific community via enabling the construction of RNA riboswitches and aptamers to treat splicing mutations, regulate mammalian gene expression, or interfere with the splicing process as reported in the literature (An et al., 2006; Beilstein et al., 2015; Berens et al., 2015; Mathur et al., 2017; Vogel et al., 2018; Mol et al., 2019; Spöring et al., 2020). Alternatively, targeting the spliceosomes, their components, and/or their mechanisms of action can be a potential treatment approach (Eymin, 2020). Designing various types of synthetic RNA-based nanodevices along with their current progression and applications as post-transcriptional modulators were discussed in a recent review (Kawasaki et al., 2020). However, these studies collectively dealt with different types of splicing patterns while neglecting the significance and complexity of the IR defects.

**FIGURE 2:**
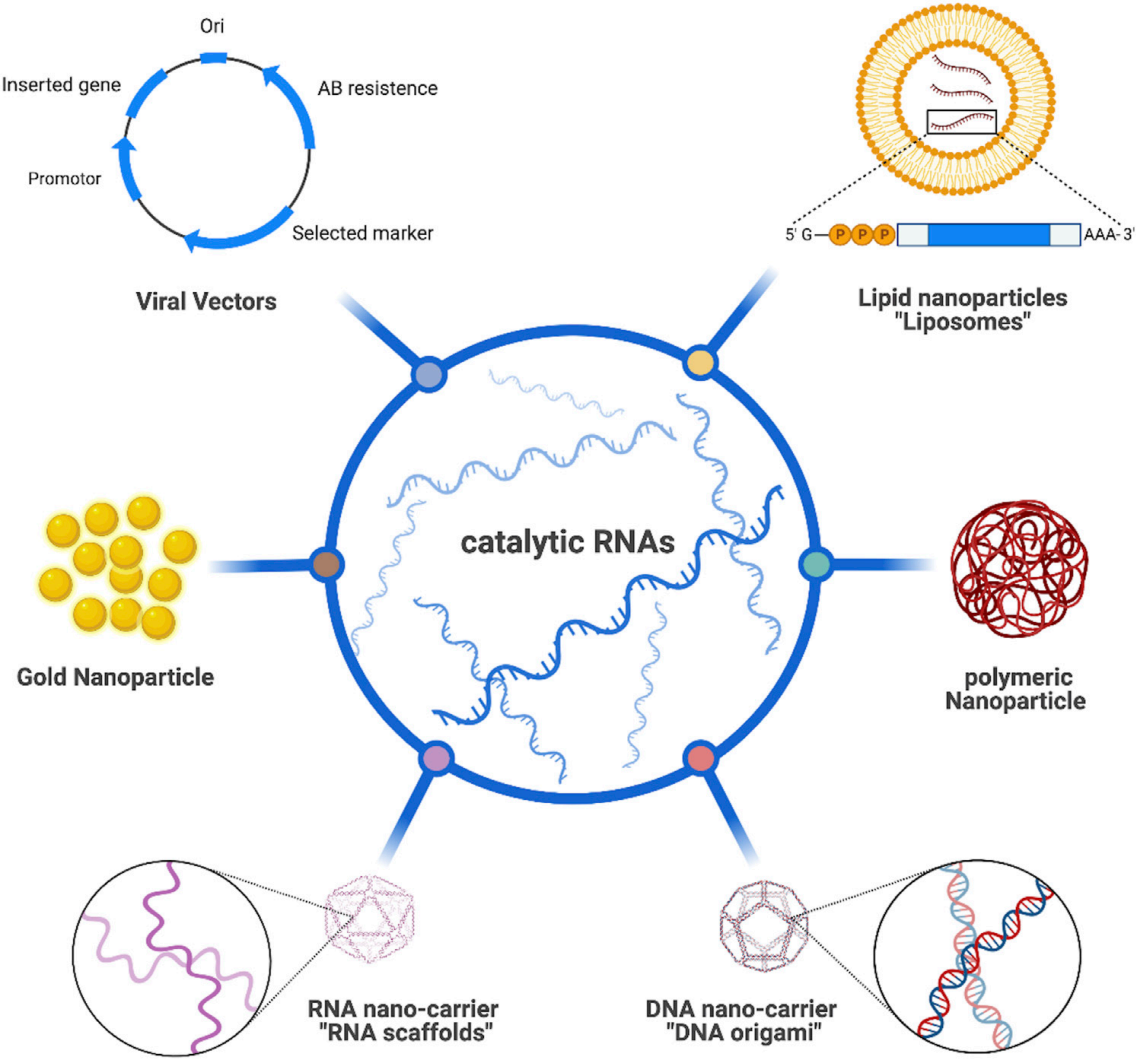
Different route to deliver catalytic RNAs.

RNA-based therapeutic platforms offer great potential in the treatment of various diseases including cancer (Lin et al., 2020). However, such platforms are still falling behind as clinical trials remain pending owing to the short life span of RNAs, their sensitivity to enzymatic degradation, and obstructed cellular internalization as a result of having a highly negatively charged backbone (Reischl and Zimmer, 2009). Scientists have extensively investigated possible routes to tackle those challenges and enhance the delivery of such therapies (Figure 2). Viral vectors are one of the potential carrier systems to deliver nucleic acid therapies providing both accuracy and protection against any enzymatic degradation of the loaded genetic materials. Despite their great properties, there are major drawbacks associated with the use of viral-based systems including their insufficiency in delivering the therapeutic agents to specific organs, which might provoke the immune system and possibly cause carcinogenesis among many other safety concerns (Lukashev and Zamyatnin, 2016; Zhou et al., 2020).

Alternatively, a more biologically feasible and safer route utilizing non-viral vectors has been used to overcome such drawbacks. Diverse types of non-viral vectors like lipid-based nanocarriers (Tagami et al., 2011; Xue et al., 2015), plasmid DNAs (Charoenphol and Bermudez, 2014; Hu et al., 2018), scaffolds (Chen et al., 2018; Kelly et al., 2019) and AuNPs (Guo et al., 2015) have been extensively studied to load and deliver RNA to the desired tissues. Lipid-based nanocarriers have numerous types but the most studied one is liposomes owing to their advantageous properties like biocompatibility, simple preparation, ease of surface modification to increase tissue targeting, and high RNA encapsulation efficiency when employing positively charged lipids. Inoh et al. (2011) successfully loaded siRNA into liposomes containing vectors and observed rapid and direct delivery to cytosol, minimal cytotoxicity, effective gene silencing, and less risk in triggering the immune response. Polymers have also attained a great attention as candidate carriers for RNA therapeutics due to their interesting properties in terms of biodegradation, cellular internalization, and the ability to control the release of loaded materials. Potential RNA polymeric carriers are countless and fully discussed in a recently published review (Ulkoski et al., 2019). Interestingly, DNA nanostructures such as DNA origami have emerged as a promising technology for delivering various bioactive molecules owing to their disintegrated internal aqueous nature allowing the delivery of different hydrophilic cargos like RNA molecules with reduced immune response and increased cellular accumulation. Lee et al. (2012) were able to construct DNA tetrahedra loaded with therapeutic siRNA allowing for ultimate and efficient therapeutic delivery. When incubated with human cervical cancer HeLa cells, gene silencing and tumor size reduction were both observed.

Following the pioneer studies conducted on DNA nanotechnology, scientists have been attracted to RNA nanotechnology via designing and building RNA nanostructures that can be applicable in the field of nanobiomedicine. RNA scaffolds emerge upon folding RNA strands into desired structures (Afonin et al., 2010; Afonin et al., 2012; Afonin et al., 2014; Sachdeva et al., 2014; Myhrvold and Silver, 2015; Bui et al., 2017; Ohno et al., 2019) as successfully did so Høiberg et al. (2019) with entrapping intrinsic siRNAs for efficient gene knockdown. Recently, metal-organic frameworks (MOFs) have been developed as nanoscale carrier systems for RNA therapies. The conducted study developed a unique UiO-NMOF exhibiting a characteristic surface morphology for co-delivering chemotherapeutic agent cisplatin and siRNA, the results shows a promising MDR gene silencing in ovarian cancer as well as the enhancement of cisplatin efficacy (He et al., 2014). Among all nanocarriers, AuNPs have been the stellar candidate for various biomedical and clinical applications including RNA therapeutics. They possess distinctive physiochemical, biological, and optical detection properties. In addition to their unique surface plasmon resonance, reduced cytotoxicity upon surface modifications with targeting ligands provides a selective and effective delivery system (Pissuwan et al., 2011; Conde et al., 2012; Guo et al., 2016; Graczyk et al., 2020). Intriguingly, developed a novel nanozyme system in which gold nanoparticles (AuNPs) were coupled with two different enzymes; natural (ligating) RNA ligase (RtcB) used to join exons after cleavage and synthetic (cleaving) DNAzyme to recognize the intron and initiate the splicing reaction. Results showed that the nanozyme selectively spliced 19 nucleotides out of RNA with 10% yield. Moreover, increased splicing reaction up to 66% was observed upon the addition of an excess amount of RNA ligase. However, the low RtcB copy number on individual AuNPs limits the splicing efficiency (Petree et al., 2018).

## Perspective and discussion

Nanotechnology can be a powerful weapon to secure healthy aging via repairing DNA and RNA damages. Life extension of patients suffering from muscular dystrophy (MD), Alzheimer disease (AD), Huntington disease (HD), or myelodysplastic syndromes (MDS) can be feasible through nanotechnology by which engineered nanorobots can perform cellular level surgeries such as splicing with high precision. To the best of our knowledge, no reports have shown artificial *in vivo* splicing in humans or even in animal models despite the advancement in performing *in vivo* RNA therapies in humans. Revolutionized gene editing tools like RNAi modalities (e.g., siRNA and miRNA) and CRISPR-Cas9 were encapsulated inside nanocarrier systems like lipid, organic, or inorganic NPs and have been used in humans to inhibit gene mutation and increase or correct gene expression (Hu et al., 2020).

Recently, United States Food and Drug Administration (USFDA) and European Commission (EC) have approved the first RNAi based therapy for clinical purposes called ONPATTRO (Patisiran), commercialized as a drug product to treat patients suffering from polyneuropathy, which is one of the symptoms associated with transthyretin amyloidosis (ATTR). A mutation in the gene coding for hereditary transthyretin (TTR), which is a protein synthesized mainly in the liver and responsible for carrying vitamin A and Thyroxine, causes protein misfolding and aggregation leading to the accumulation of formed amyloid at different locations and hence developing ATTR accompanied with several manifestations including polyneuropathy. ONPATTRO is produced using lipid NPs to encapsulate siRNA and enhance its delivery to the hepatocytes, thereby inhibiting the gene expression of both wild and mutant types (Huang, 2019). Similarly, Gillmore and colleagues investigated the effect of CRISPR-Cas9 as a potential therapeutic agent to knockdown TTR protein. Clinical and *in-vivo* results conducted on a small group of ATTR patients suffering from polyneuropathy showed a durable inhibition of TTR gene expression ranging from 80%–90% after 28 days of receiving a single dose (Gillmore et al., 2021). New advancement and alteration to CRISPER utilized novel base switchers known as base editing where Cas9 nickase is coupled to deaminase protein to allow single base conversions. Such advancements can potentially improve the use of gene editing technologies as treatment interventions for many alternative splicing defects. Chemello et al. (2021) reported combined two different strategies; namely, base and prime editing to developed gene editing tool to modify dystrophin gene where mutation in the splice donor leads to exon 51 deletion causing Duchenne muscular dystrophy (DMD). Their finding demonstrates a successful correction of the exon deletion of DMD gene tested on human cardiac iPSC models of DMD patients. Interestingly, have exploited a unique base editing approach to disrupt genes and minimize the unwanted double stranded breaks that Cas9 usually rely on to edit genes. They introduced their SpliceR tool to design base edited sgRNA to target splice site and achieved more reliable and efficient effect in primary human T cells (Kluesner et al., 2021). Similarly, investigators have employed CRISPR-cas9 base editing techniques targeting splice acceptor site to achieve a permanent exon skipping and improve the compatibility with adeno-associated viral packaging for *in-vivo* treatment (Winter et al., 2019).

Givosiran is another example of the developed RNAi based therapeutic agent loaded in lipid NPs to reduce the expression of delta aminolevulinic acid synthase 1 (ALAS1) gene and hence treating acute Intermittent porphyria (AIP). Overexpression of ALAS1 could lead to the deposition of neurotoxic heme compounds leading to painful neurovisceral attacks or causing chronic symptoms. Promising and effective reduction in the level of porphyria attacks was observed in clinical trials following the administration of Givosiran to AIP patients (Yasuda et al., 2014; Balwani et al., 2019; Sardh et al., 2019; Agarwal et al., 2020; Balwani et al., 2020; de Paula Brandão et al., 2020). Such findings emphasize the critical role of lipid NPs in accelerating the clinical use of RNA therapies. During the COVID-19 pandemic, the rapid response from Pfizer-BioNTech and Moderna by exploiting lipid NPs to encapsulate mRNA helped in developing the vaccine, which was granted the emergency authorization by the USFDA to combat against the emerging SARS-CoV-2 virus (Milane and Amiji, 2021).

Despite the great potency of using RNA therapies, a number of concerns need to be raised and tackled. For instance, RNAs degradation by endosomes and lysosomes must be avoided for the successful translocation to cytoplasm wherein selective targeting might occur. Wang et al. developed a novel endoplasmic reticulum membrane-modified hybrid nanoplexes (EhCv/siRNA NPs) encapsulating siRNA and protecting it from lysosomal degradation for efficient siRNA transportation to cytoplasm in order to improve siRNA silencing ability (Qiu et al., 2019). Nanocarriers of lipids and lipidoids require specific structural design criteria including selected phospholipids exhibiting two or more hydrophobic tails, tertiary amines, lipidoid O13 tail, and a pKa value ≥ 5.5, in order to mediate the selective RNA delivery to target tissues (Whitehead et al., 2014). Generally, RNA oligonucleotides are known to exhibit lower stability in cellular environments. Therefore, characteristic chemical modifications can be used to increase the stability and efficacy of RNAs. Recent study investigated the addition of (E)- 7 vinyl phosphonate moiety at the 5’ end of the oligonucleotide to enhance the stability of siRNA (Parmar et al., 2018). Another essential requirement for the carrier system is the ability to complex with RNAi material to enhance the payload concentration (Dong et al., 2019). The recent advancement in self-targeting NPs to selective organs (Mohammadinejad et al., 2020; Alsudir et al., 2021) could be advantageous to enhance the delivery of RNA therapies boosting their medicinal efficacy and accelerating their translation into clinics.
